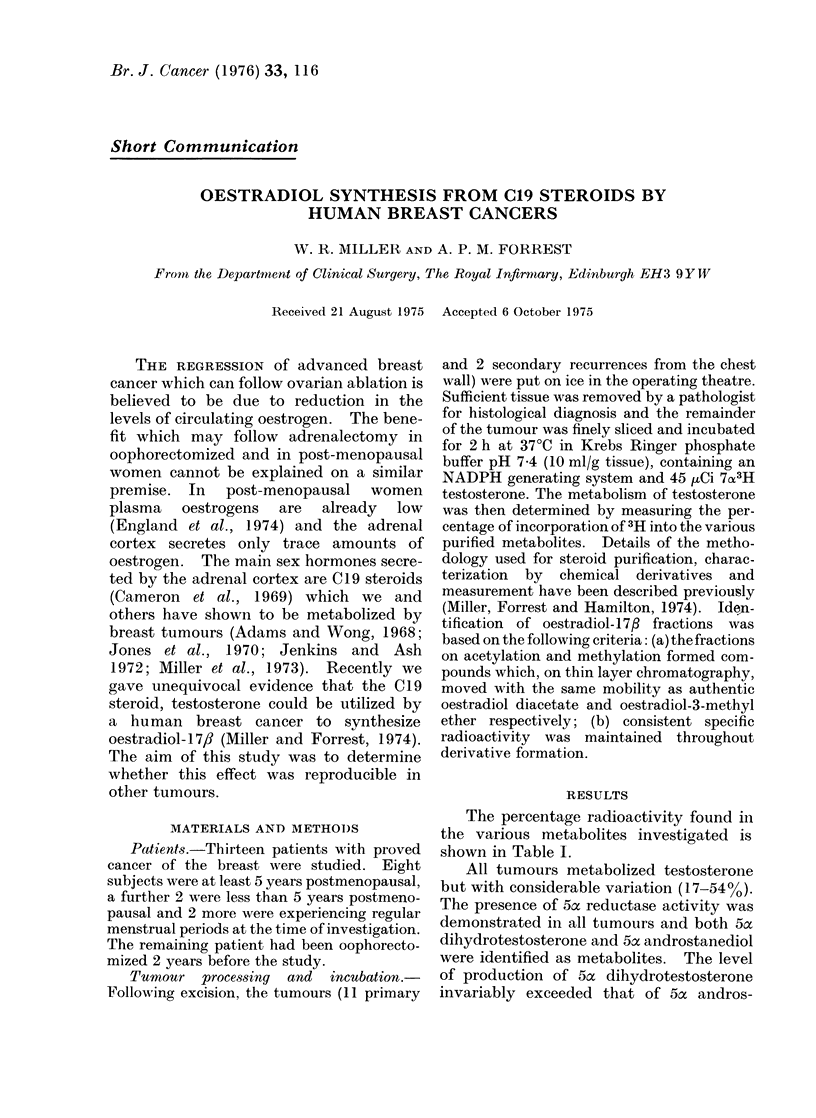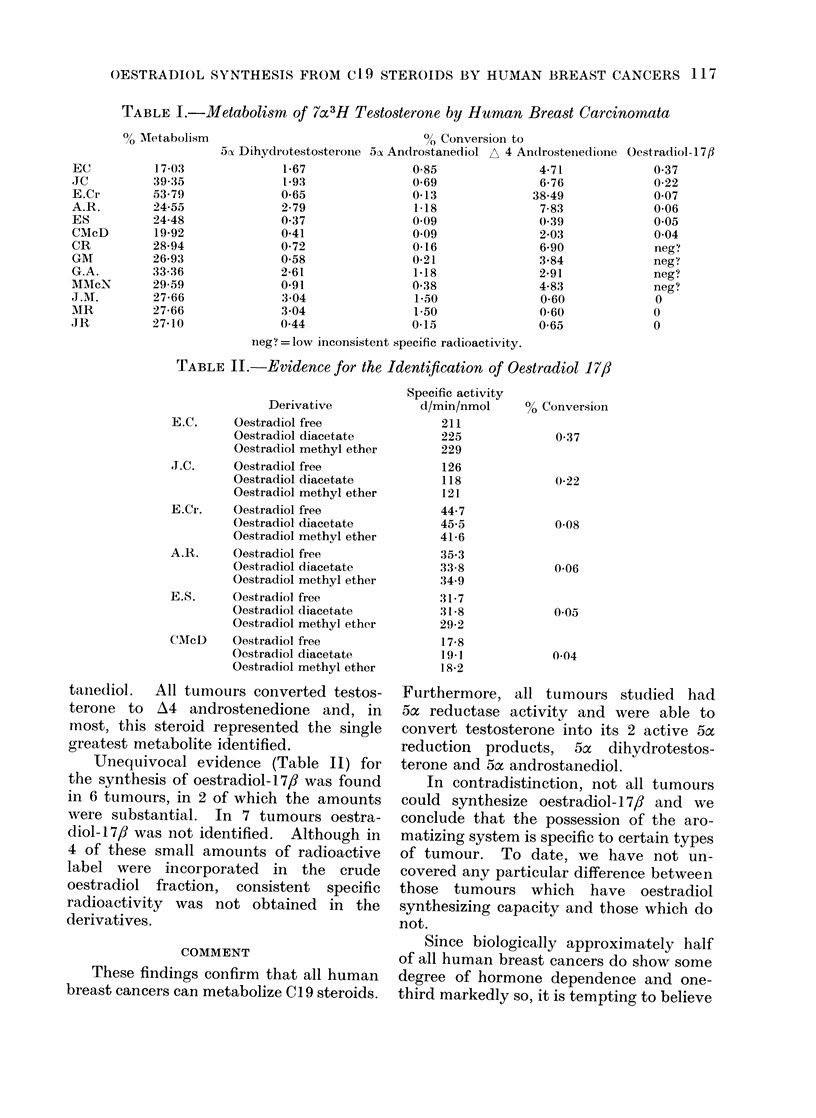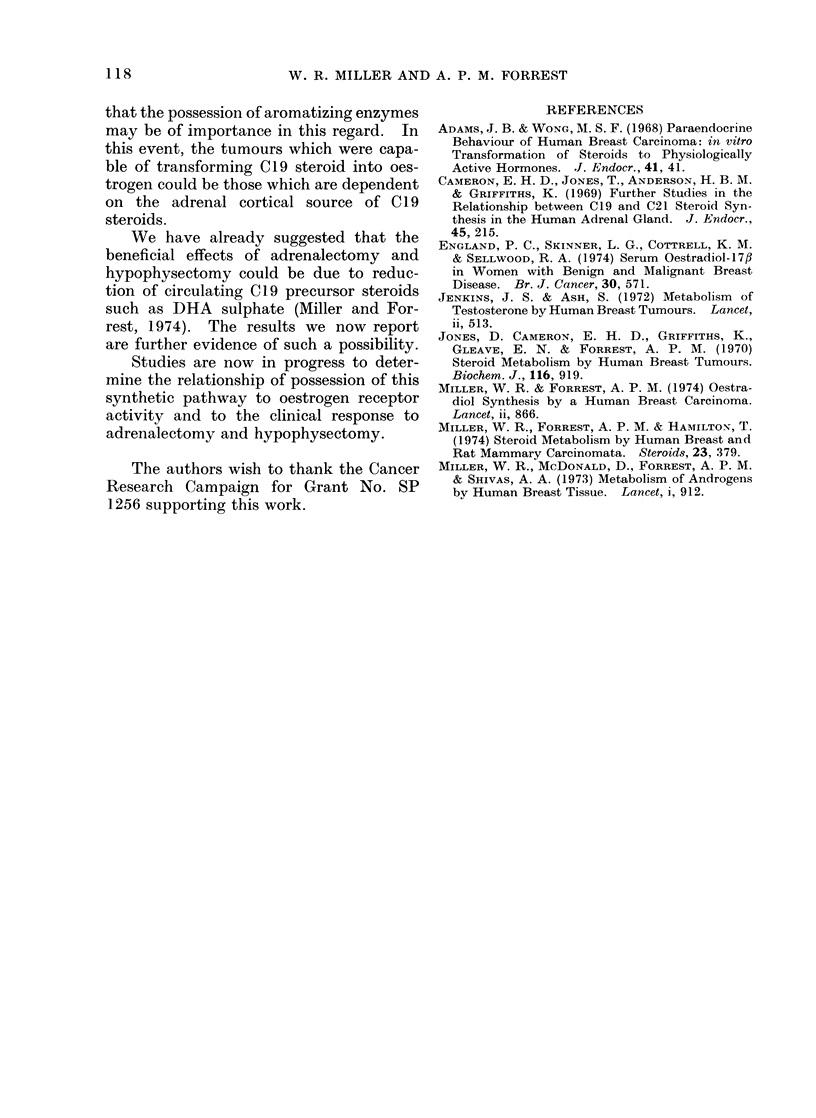# Oestradiol synthesis from C19 steroids by human breast cancers.

**DOI:** 10.1038/bjc.1976.13

**Published:** 1976-01

**Authors:** W. R. Miller, A. P. Forrest


*Br. J. Cancer* (1976) **33,** 116

### *Short Communication*

### OESTRADIOL SYNTHESIS FROM C19 STEROIDS BY HUMAN BREAST CANCERS

W. R. MILLER AND A. P. M. FORREST

*From the Department of Clinical Surgery, The Royal Infirmary, Edinburgh EH3 9YW*

Received 21 August 1975 Accepted 6 October 1975

THE REGRESSION of advanced breast cancer which can follow ovarian ablation is believed to be due to reduction in the levels of circulating oestrogen. The benefit which may follow adrenalectomy in oophorectomized and in post-menopausal women cannot be explained on a similar premise. In post-menopausal women plasma oestrogens are already low (England *et al.*, 1974) and the adrenal cortex secretes only trace amounts of oestrogen. The main sex hormones secreted by the adrenal cortex are C19 steroids (Cameron *et al.*, 1969) which we and others have shown to be metabolized by breast tumours (Adams and Wong, 1968; Jones *et al.*, 1970; Jenkins and Ash 1972; Miller *et al.*, 1973). Recently we gave unequivocal evidence that the C19 steroid, testosterone could be utilized by a human breast cancer to synthesize oestradiol-17$\beta$ (Miller and Forrest, 1974). The aim of this study was to determine whether this effect was reproducible in other tumours.

### MATERIALS AND METHODS

*Patients.*—Thirteen patients with proved cancer of the breast were studied. Eight subjects were at least 5 years postmenopausal, a further 2 were less than 5 years postmenopausal and 2 more were experiencing regular menstrual periods at the time of investigation. The remaining patient had been oophorectomized 2 years before the study.

*Tumour processing and incubation.*—Following excision, the tumours (11 primary and 2 secondary recurrences from the chest wall) were put on ice in the operating theatre. Sufficient tissue was removed by a pathologist for histological diagnosis and the remainder of the tumour was finely sliced and incubated for 2 h at 37°C in Krebs Ringer phosphate buffer pH 7·4 (10 ml/g tissue), containing an NADPH generating system and 45 $\mu$Ci 7$\alpha$$^3$H testosterone. The metabolism of testosterone was then determined by measuring the percentage of incorporation of $^3$H into the various purified metabolites. Details of the methodology used for steroid purification, characterization by chemical derivatives and measurement have been described previously (Miller, Forrest and Hamilton, 1974). Identification of oestradiol-17$\beta$ fractions was based on the following criteria: (a) the fractions on acetylation and methylation formed compounds which, on thin layer chromatography, moved with the same mobility as authentic oestradiol diacetate and oestradiol-3-methyl ether respectively; (b) consistent specific radioactivity was maintained throughout derivative formation.

### RESULTS

The percentage radioactivity found in the various metabolites investigated is shown in Table I.

All tumours metabolized testosterone but with considerable variation (17–54%). The presence of 5$\alpha$ reductase activity was demonstrated in all tumours and both 5$\alpha$ dihydrotestosterone and 5$\alpha$ androstanediol were identified as metabolites. The level of production of 5$\alpha$ dihydrotestosterone invariably exceeded that of 5$\alpha$ andros-

TABLE I.—*Metabolism of 7α³H Testosterone by Human Breast Carcinomata*

| | % Metabolism | % Conversion to | | | |
|---|---|---|---|---|---|
| | | 5α Dihydrotestosterone | 5α Androstanediol | △ 4 Androstenedione | Oestradiol-17β |
| EC | 17·03 | 1·67 | 0·85 | 4·71 | 0·37 |
| JC | 39·35 | 1·93 | 0·69 | 6·76 | 0·22 |
| E.Cr | 53·79 | 0·65 | 0·13 | 38·49 | 0·07 |
| A.R. | 24·55 | 2·79 | 1·18 | 7·83 | 0·06 |
| ES | 24·48 | 0·37 | 0·09 | 0·39 | 0·05 |
| CMcD | 19·92 | 0·41 | 0·09 | 2·03 | 0·04 |
| CR | 28·94 | 0·72 | 0·16 | 6·90 | neg? |
| GM | 26·93 | 0·58 | 0·21 | 3·84 | neg? |
| G.A. | 33·36 | 2·61 | 1·18 | 2·91 | neg? |
| MMcN | 29·59 | 0·91 | 0·38 | 4·83 | neg? |
| J.M. | 27·66 | 3·04 | 1·50 | 0·60 | 0 |
| MR | 27·66 | 3·04 | 1·50 | 0·60 | 0 |
| JR | 27·10 | 0·44 | 0·15 | 0·65 | 0 |

neg? = low inconsistent specific radioactivity.

TABLE II.—*Evidence for the Identification of Oestradiol 17β*

| | Derivative | Specific activity d/min/nmol | % Conversion |
|---|---|---|---|
| E.C. | Oestradiol free | 211 | |
| | Oestradiol diacetate | 225 | 0·37 |
| | Oestradiol methyl ether | 229 | |
| J.C. | Oestradiol free | 126 | |
| | Oestradiol diacetate | 118 | 0·22 |
| | Oestradiol methyl ether | 121 | |
| E.Cr. | Oestradiol free | 44·7 | |
| | Oestradiol diacetate | 45·5 | 0·08 |
| | Oestradiol methyl ether | 41·6 | |
| A.R. | Oestradiol free | 35·3 | |
| | Oestradiol diacetate | 33·8 | 0·06 |
| | Oestradiol methyl ether | 34·9 | |
| E.S. | Oestradiol free | 31·7 | |
| | Oestradiol diacetate | 31·8 | 0·05 |
| | Oestradiol methyl ether | 29·2 | |
| CMcD | Oestradiol free | 17·8 | |
| | Oestradiol diacetate | 19·1 | 0·04 |
| | Oestradiol methyl ether | 18·2 | |

tanediol. All tumours converted testosterone to Δ4 androstenedione and, in most, this steroid represented the single greatest metabolite identified.

Unequivocal evidence (Table II) for the synthesis of oestradiol-17β was found in 6 tumours, in 2 of which the amounts were substantial. In 7 tumours oestradiol-17β was not identified. Although in 4 of these small amounts of radioactive label were incorporated in the crude oestradiol fraction, consistent specific radioactivity was not obtained in the derivatives.

### COMMENT

These findings confirm that all human breast cancers can metabolize C19 steroids. Furthermore, all tumours studied had 5α reductase activity and were able to convert testosterone into its 2 active 5α reduction products, 5α dihydrotestosterone and 5α androstanediol.

In contradistinction, not all tumours could synthesize oestradiol-17β and we conclude that the possession of the aromatizing system is specific to certain types of tumour. To date, we have not uncovered any particular difference between those tumours which have oestradiol synthesizing capacity and those which do not.

Since biologically approximately half of all human breast cancers do show some degree of hormone dependence and one-third markedly so, it is tempting to believe that the possession of aromatizing enzymes may be of importance in this regard. In this event, the tumours which were capable of transforming C19 steroid into oestrogen could be those which are dependent on the adrenal cortical source of C19 steroids.

We have already suggested that the beneficial effects of adrenalectomy and hypophysectomy could be due to reduction of circulating C19 precursor steroids such as DHA sulphate (Miller and Forrest, 1974). The results we now report are further evidence of such a possibility.

Studies are now in progress to determine the relationship of possession of this synthetic pathway to oestrogen receptor activity and to the clinical response to adrenalectomy and hypophysectomy.

The authors wish to thank the Cancer Research Campaign for Grant No. SP 1256 supporting this work.

### REFERENCES

ADAMS, J. B. & WONG, M. S. F. (1968) Paraendocrine Behaviour of Human Breast Carcinoma: *in vitro* Transformation of Steroids to Physiologically Active Hormones. *J. Endocr.*, **41**, 41.

CAMERON, E. H. D., JONES, T., ANDERSON, H. B. M. & GRIFFITHS, K. (1969) Further Studies in the Relationship between C19 and C21 Steroid Synthesis in the Human Adrenal Gland. *J. Endocr.*, **45**, 215.

ENGLAND, P. C., SKINNER, L. G., COTTRELL, K. M. & SELLWOOD, R. A. (1974) Serum Oestradiol-17$\beta$ in Women with Benign and Malignant Breast Disease. *Br. J. Cancer*, **30**, 571.

JENKINS, J. S. & ASH, S. (1972) Metabolism of Testosterone by Human Breast Tumours. *Lancet*, ii, 513.

JONES, D. CAMERON, E. H. D., GRIFFITHS, K., GLEAVE, E. N. & FORREST, A. P. M. (1970) Steroid Metabolism by Human Breast Tumours. *Biochem. J.*, **116**, 919.

MILLER, W. R. & FORREST, A. P. M. (1974) Oestradiol Synthesis by a Human Breast Carcinoma. *Lancet*, ii, 866.

MILLER, W. R., FORREST, A. P. M. & HAMILTON, T. (1974) Steroid Metabolism by Human Breast and Rat Mammary Carcinomata. *Steroids*, **23**, 379.

MILLER, W. R., MCDONALD, D., FORREST, A. P. M. & SHIVAS, A. A. (1973) Metabolism of Androgens by Human Breast Tissue. *Lancet*, i, 912.